# Inhibition of Autophagy Promotes the Anti-Tumor Effect of Metformin in Oral Squamous Cell Carcinoma

**DOI:** 10.3390/cancers14174185

**Published:** 2022-08-29

**Authors:** Wei Zhao, Chen Chen, Jianjun Zhou, Xiaoqing Chen, Kuan Cai, Miaomiao Shen, Xuan Chen, Lei Jiang, Guodong Wang

**Affiliations:** 1 Department of Stomatology, Changzheng Hospital, Naval Medical University, Shanghai 200003, China; 2 Department of Stomatology, 900 Hospital of the Joint Logistics Team, Fuzhou 350025, China

**Keywords:** metformin, hydroxychloroquine, oral squamous cell carcinoma, apoptosis, autophagy

## Abstract

**Simple Summary:**

In this study, we found that metformin could inhibit the cell proliferation of Oral Squamous Cell Carcinoma (OSCC) by promoting apoptosis and blocking the cell cycle. Furthermore, inhibiting autophagy with hydroxychloroquine (HCQ) could enhance the anti-tumor effect of metformin in OSCC.

**Abstract:**

Oral Squamous Cell Carcinoma (OSCC) is the most common malignant tumor in the head and neck. Due to its high malignancy and easy recurrence, the five-year survival rate is only 50–60%. Currently, commonly used chemotherapy drugs for OSCC include cisplatin, paclitaxel, and fluorouracil, which are highly cytotoxic and cause drug resistance in patients. Therefore, a safe and effective treatment strategy for OSCC is urgent. To address this issue, our study investigated the anti-tumor activity of metformin (the first-line diabetes drug) in OSCC. We found that metformin could inhibit OSCC cell proliferation by promoting apoptosis and blocking the cell cycle in G1 phase. Additionally, we also found that metformin could induce protective autophagy of OSCC cells. After inhibiting autophagy with hydroxychloroquine (HCQ), the metformin-induced apoptosis was enhanced. In vitro, metformin inhibited the growth of subcutaneous xenograft tumor in nude mice and HCQ enhanced this effect of metformin. Therefore, metformin combined with HCQ may become a safe and effective treatment strategy for OSCC.

## 1. Introduction

Tongue, gums, and buccal mucosa are the common sites of Oral Squamous Cell Carcinoma (OSCC), which is the most common head and neck squamous cell carcinoma [1]. The main affected population of OSCC is adult males over 50 years old and smoking, drinking, betel nut chewing and HPV infection are closely related to the development of OSCC [2]. The main OSCC characteristics are high invasiveness, high malignancy, and poor prognosis [3]. In 2020, 380,000 new cases and 180,000 deaths of OSCC were reported globally [4]. At present, surgical treatment, radiotherapy and chemotherapy have made significant breakthroughs, but the 5-year average survival rates of OSCC remain at a poor level, of only 50–60% [5]. There is an urgent need for a safe and effective treatment strategy for OSCC.

Metformin, a biguanide derivative extracted from *Galega Officinalis* (French lilac), has been the first-line treatment of diabetes for more than 60 years [6]. Metformin exerts hypoglycemic effects, mainly by reducing hepatic gluconeogenesis and enhancing peripheral glucose uptake [7]. Recently, more and more studies have shown that metformin has preventive and therapeutic effects on various tumors. In some basic studies, researchers found that metformin could inhibit the proliferation of different tumor cells, including lung cancer, liver cancer and breast cancer [8,9,10]. Additionally, metformin can act as an adjuvant therapy to enhance the efficacy of other anti-tumor treatments [11]. The results of epidemiological studies have shown that metformin can reduce cancer incidence and cancer-related mortality in patients with diabetes [12]. Therefore, metformin may become a potential anti-tumor drug. The anti-tumor mechanisms of metformin are categorized into two types: the indirect effect and the direct effect. The indirect impact was caused by metformin decreasing systemic glucose and insulin levels. Metformin may also directly affect tumor cells through the activation of AMPK-dependent or AMPK-independent signaling pathways [13]. Wang Ying et al. have reported that metformin can inhibit proliferation of OSCC cells [14], but the potential mechanism, and how to enhance the curative effect, of metformin in OSCC remain to be investigated.

Programmed Cell Death (PCD) is the orderly and autonomous death of cells controlled by genes [15], which mainly includes apoptosis and autophagy [16]. The cells respond to different stresses and death stimulation by apoptosis and autophagy to maintain intracellular homeostasis [17]. Apoptosis is the most thoroughly studied form of PCD, which can be triggered by diverse intracellular and extracellular factors. The intracellular apoptosis factors include high levels of Reactive Oxygen Species (ROS), increased intracellular calcium concentration, and DNA damage. The growth factors, namely, nitric oxide, toxins and bacterial pathogens, are the main extrinsic apoptosis factors [18]. Numerous morphological changes occur when cells undergo apoptosis, including nuclear fragmentation, chromatin condensation, cell shrinkage, and apoptotic body formation [19]. Autophagy is a lysosome-mediated cellular degradation pathway in eukaryotic cells [20]. Autophagy ensures organelle renewal, cellular metabolism, and intracellular homeostasis by removing intracellular damaged organelles, denatured proteins and invading pathogens [21]. Autophagy has different effects in different stages of cancer. In some circumstances, autophagy can inhibit tumor progression [22]. While in others, autophagy can facilitate tumor progression by inhibiting apoptosis [23]. In addition, after the autophagy is inhibited, the chemosensitivity of tumor cells is restored [24,25]. Apoptosis and autophagy in tumor cells have a complex relationship, and the underlying molecular interactions between them remain elusive.

Our study showed that metformin could inhibit the proliferation, migration, and invasion of OSCC, and could promote OSCC cell apoptosis and autophagy. Furthermore, the inhibition of autophagy by hydroxychloroquine (HCQ) could enhance the apoptosis of the OSCC cells induced by metformin. Metformin and HCQ could act synergistically in vivo to exert anti-OSCC effects.

## 2. Materials and Methods

### 2.1. Cell Culture

The OSCC cell lines (CAL27, SCC9, SCC25) were obtained from ATCC (Manassas, VA, USA). CAL27 cells were cultured in DMEM high glucose medium (Gibico, Shanghai, China). SCC9 and SCC25 cells were cultured in F12 medium (Meilunebio, Dalian, China). The medium was supplemented with 10% FBS (Biolnd, Kibbutz Beit Haemek, Israel), and 1% penicillin-streptomycin solution (Gibico, Grand Island, NY, USA). The cells were incubated at 37 °C with 5% CO_2_.

### 2.2. CCK-8 Assay

The OSCC cells (5000/well) in good condition were seeded in 96-well plates. All cells were treated with the gradient concentrations of metformin (Sigma-Aldrich, St. Louis, MO, USA) for 24 h and 48 h, with or without HCQ (MedChemExpress, Monmouth Junction, NJ, USA). To detect cell viability, we performed the CCK-8 assay. In brief, the CCK-8 reagent (Dojindo, Kumamoto, Japan) was co-incubated with the cells for 1.5 h. A SpectraMax i3x microplate reader (Molecular Devices, Sunnyvale, CA, USA) was used to measure the absorbance at 450 nm.

### 2.3. Colony Formation Assay

The OSCC cells (200/well) in good condition were seeded in 6-well plates. All cells were incubated with metformin (0, 12, 24 mM) for 48 h after completely adhering to the plates. After 48 h, the medium containing metformin was replaced by normal medium and the cells were cultured for a further 12 days. All cells were fixed with paraformaldehyde, followed by being stained with crystal violet.

### 2.4. Scratch Assay

CAL27 cells were seeded in a 6-well plate and cultured overnight. When the cell confluency reached 100%, the 20 μL tips were used to make scratches on the cell surface. The scratches were photographed with an inverted microscope (CKX31, Olympus, Tokyo, Japan). Then, cells were treated with metformin (0, 12, 24 mM) dissolved in serum-free medium for 24 h and 48 h. The scratches at the same location were photographed by microscope. Image j was used to measure the area of scratches.

### 2.5. Transwell Invasion Assay

The upper transwell chamber (8-μm; Corning, Corning, NY, USA) was covered with 20 μL diluted Matrigel (dilution ratio: 1:8, Corning) and incubated in the incubator overnight until the Matrigel solidified. CAL27 cells were serum-starved for 12 h. Then cells (2 × 10^5^/well) were seeded in the upper transwell chamber with metformin (0, 12, 24 mM) dissolved in serum-free medium for 48 h. The lower chamber was filled with 600 μL medium containing serum. After 48 h, all cells were fixed with paraformaldehyde, followed by being stained with crystal violet. Six fields of view were selected randomly to take pictures.

### 2.6. Apoptosis Assay

The OSCC cells (2.5 × 10^5^/well) in good condition were seeded in 6-well plates. Then the cells were exposed to metformin (0, 12, 24 mM) for 48 h, with or without HCQ (20 μM). We used an Annexin V-FITC Apoptosis Detection kit (BD Biosciences, San Jose, CA, USA) to detect the apoptosis level. According to the instructions, all cells were harvested, followed by being washed with PBS. Then, 1 X binding buffer was used to resuspend the cells. The cells were incubated with Annexin V-FITC and PI in the dark. After incubation, all samples were immediately analyzed by flow cytometry (CyAN ADP, Beckman-Coulter, Miami, FL, USA).

### 2.7. Cell Cycle Assay

The OSCC cells (2.5 × 10^5^/well) in good condition were seeded in 6-well plates. Then, the cells were exposed to metformin (0, 12, 24 mM) for 48 h. We used a cell-cycle detection kit (MultiSciences, Hangzhou, China) to detect the cell-cycle distribution. According to the instructions, cells were collected followed by being washed with PBS. DNA Staining Solution (1 mL) was used to resuspend the cells and the permeabilization solution (10 μL) was added to cells. The cells were incubated in the dark. All samples were analyzed by flow cytometry.

### 2.8. ROS Assay

The ROS assay kit (Beyotime, Shanghai, China) was used to measure the ROS level. CAL27 cells (2.5 × 10^5^/well) in good condition were seeded in 6-well plates. Then, the cells were exposed to metformin (0, 12, 24 mM) for 48 h. The cells were incubated with DCFH-DA for 20 min. Finally, the ROS was analyzed by a flow cytometer.

### 2.9. MRFP-GFP-LC3 Analysis

CAL27 cells (2.5 × 10^5^/well) were seeded in 24 well-plates placed with cell-climbing slices and cultured overnight. Then, the cells were treated with metformin (0, 12, 24 mM) for 48 h. At the same time, CAL27 cells were transfected with the mRFP-GFP-LC3 adenovirus. All cells were washed with PBS, followed by being fixed with formaldehyde. Then, cells were stained with DAPI. A confocal laser scanning microscope (Olympus, Tokyo, Japan) was used to observe the autophagic flux of CAL27 cells. Red dots represent autophagolysosomes, and yellow dots represent autophagosomes.

### 2.10. Transmission Electron Microscopy (TEM)

The autophagosomes and nuclei in CAL27 cells were observed by the TEM (Hitachi, Tokyo, Japan). CAL27 cells were exposed to metformin (0, 12, 24 mM) for 48 h. All cells were collected and fixed with 2.5% glutaraldehyde overnight. Then, cells were washed with PBS and post-fixed with 1% OsO4 aqueous solution. Then, cells were dehydrated with the gradient concentrations of ethanol and replaced with the gradient concentrations of acetone. The cells were embedded into epoxy resin for sections (70 nm). The sections were stained with uranyl acetate and lead citrate. Finally, all samples were observed by TEM.

### 2.11. Western Blot

CAL27 cells were exposed to metformin (0, 12, 24 mM), with or without HCQ (20 μM), for 48 h. A mixture of RIPA lysate (Epizyme, Shanghai, China) and protease inhibitor (Epizyme, Shanghai, China) was used to extract proteins in the CAL27 cells. The Protein lysates were centrifuged at 12,000 rpm/min for 20 min at 4 °C, and the supernatant was collected. The concentration of proteins was detected by a BCA kit (Beyotime, Shanghai, China). The proteins and SDS loading buffer were mixed and boiled at 100 °C for 20 min. The samples were added into SDS-PAGE for separation, followed by being transferred into the PVDF membranes. The membranes were blocked with 5% skim milk powder for 1 h at room temperature. Then, the membranes were incubated overnight at 4 °C with primary antibody, which included the following: P21 (1:1000, Abcam, ab109520, Cambridge, MA, USA), Cyclin D1(1:1000, Abcam, ab134175), Cleaved-caspase3(1:1000, Abcam, ab32042), Bcl-2(1:1000, Abcam, ab32124), Bax(1:1000, Abcam, ab32503), LC3(1:1000, CST, #12741, Danvers, MA, USA), P62(1:1000, Abcam, ab207305), Beclin-1(1:1000, Abcam, ab207612), GAPDH(1:1000, Affinity, AF7021, Jiangsu, China). The next day, the membranes were washed with TBST three times and incubated with goat anti-rabbit IgG (1:10,000, CST, #5366) for 2 h. Finally, the membranes were visualized with Odyssey CLx imager (Odyssey V3.0). The Supplementary Materials include the Original Images for Blots/Gels (Figure S4).

### 2.12. Tumor Xenograft Studies

The anti-OSCC activity of metformin and HCQ in vivo was explored with animal experiments. The ethics committees of the Naval Medical University approved the whole experiment. Female nude mice (5–6 weeks old) were purchased from Jihui Laboratory Animal Care (Shanghai, China) and fed with sterile water and sterile feed. CAL27 cells (1 × 10^7^) were injected into the subcutaneous area of the nude mice. After the tumor volume of mice reached 100 mm^3^, all mice were randomly divided into four groups: PBS group, metformin group (250 mg/kg/d), HCQ group (50 mg/kg/d), and the combined group. Drugs were injected intraperitoneally. The tumor volume and mice weight were measured every three days. After treatment for 18 days, all mice were sacrificed by cervical dislocation. The tumors of mice were separated from the subcutaneous area and weighed. Then the tumor tissue and main organs were immediately fixed in 4% paraformaldehyde. The toxicity of drugs to vital organs was assessed with H&E staining. The immunohistochemistry (IHC) was performed to assess the related protein expression in tumor tissue after treatment.

### 2.13. Statistical Analysis

All data were expressed as mean ± SD and analyzed by ANOVA. * *p* < 0.05, ** *p* < 0.01 and *** *p* < 0.001 were considered statistically significant.

## 3. Results

### 3.1. Metformin Suppresses the Proliferation of OSCC Cells

**Figure 1:**
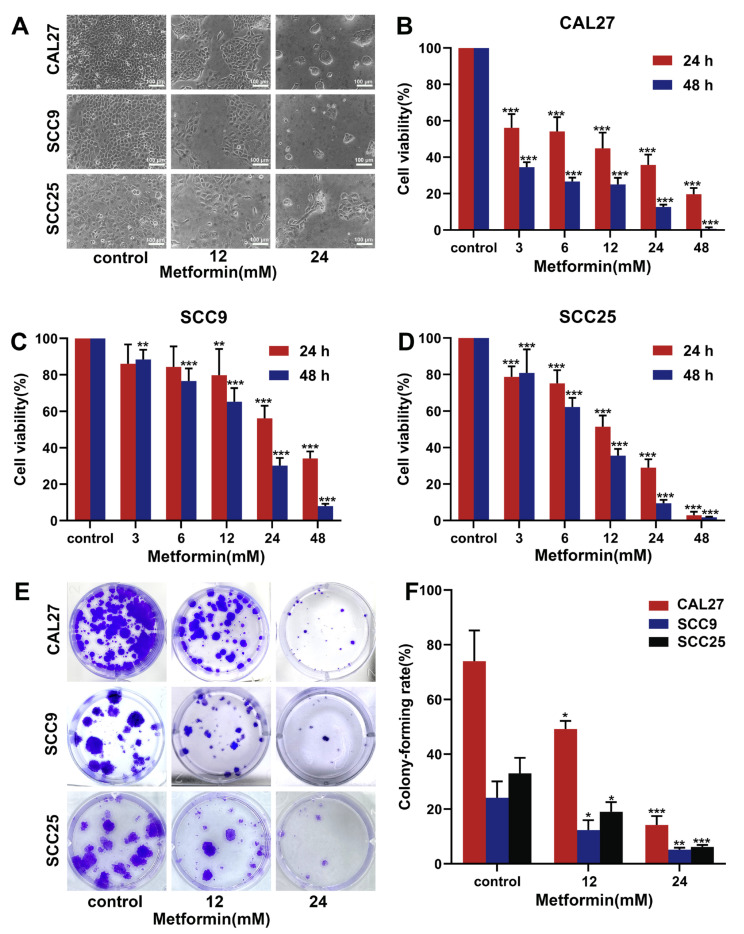
Metformin inhibited the ability of proliferation and colony-forming in OSCC cells. (**A**). The OSCC cell lines (CAL27, SCC9, SCC25) were incubated with different concentrations of metformin for 24 h, and then the cell morphology was observed by the inverted microscopy. The magnification = 100×, scale bars = 100 μm. (**B**–**D**) The OSCC cell lines were treated with the gradient concentrations of metformin for 24 h and 48 h, and then the CCK-8 assay was used to detect the cell viability. (**E**, **F**) The OSCC cell lines were treated with different concentrations of metformin for 48 h and incubated with fresh culture medium for 12 days. The cells were fixed with paraformaldehyde and stained with crystal violet. Finally, the colony-forming rate was calculated as follows: The colony-forming rate = (number of cell clones/number of seeded cells) × 100%. The data are shown in the bar graph as mean ± SD. * *p* ≤ 0.05, ** *p* ≤ 0.01 and *** *p* ≤ 0.001 versus the control group.

We performed the CCK-8 assay and colony formation assay to assess the proliferation capacity of OSCC cells. The OSCC cells treated with metformin were observed with a microscope. In the control group, the cells were arranged neatly and in the shape of cobblestones, but in the treatment group, the cell number decreased, and cell morphology was shrunken or swollen (Figure 1A). The viability of OSCC cells was significantly inhibited by metformin in a dose and time-dependent manner (Figure 1B–D). As the metformin concentration increased, the number of cell clones decreased (Figure 1E,F). Collectively, these results suggested that metformin suppressed the proliferation of OSCC cells.

### 3.2. Metformin Suppresses Migration and Invasion in OSCC Cells 

**Figure 2:**
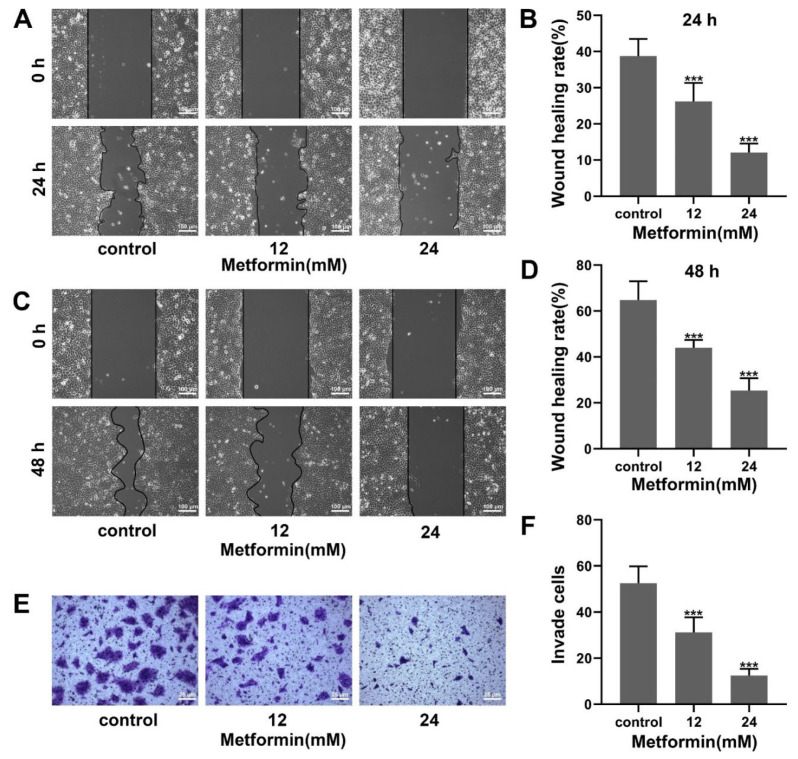
Metformin inhibited migration and invasion in OSCC cells. (**A**–**D**) The wound-scratch assay was carried out to detect the migrative ability of CAL27 cells treated with metformin for 24 h and 48 h. The wound healing rate = (scratch area at 24 h and 48 h-scratch area at 0 h/scratch area at 0 h) × 100%. The magnification = 100×, scale bars = 100 μm. (**E,F**) A transwell assay for the invasion was carried out to detect the invasive ability of CAL27 cells treated with metformin for 48 h. The magnification = 400×, scale bars = 25 μm. The data are shown in the bar graph as mean ± SD, *** *p* ≤ 0.001 versus the control group.

To assess the effect of metformin on the migration ability of OSCC cells, a scratch assay was performed. As shown in Figure 2A–D, the scratch area was increased as the concentration of metformin increased. Metformin inhibited the migration ability of CAL27 cells. To assess the effect of metformin on the invasion ability of OSCC cells, a transwell invasion assay was performed. The cell number crossing the chambers decreased with the elevation of metformin concentration (Figure 2E,F). Metformin inhibited the invasion ability of CAL27 cells. Collectively, metformin inhibited migration and invasion in OSCC cells.

### 3.3. Metformin Induces G1 Phase Cell-Cycle Block in OSCC Cells

**Figure 3:**
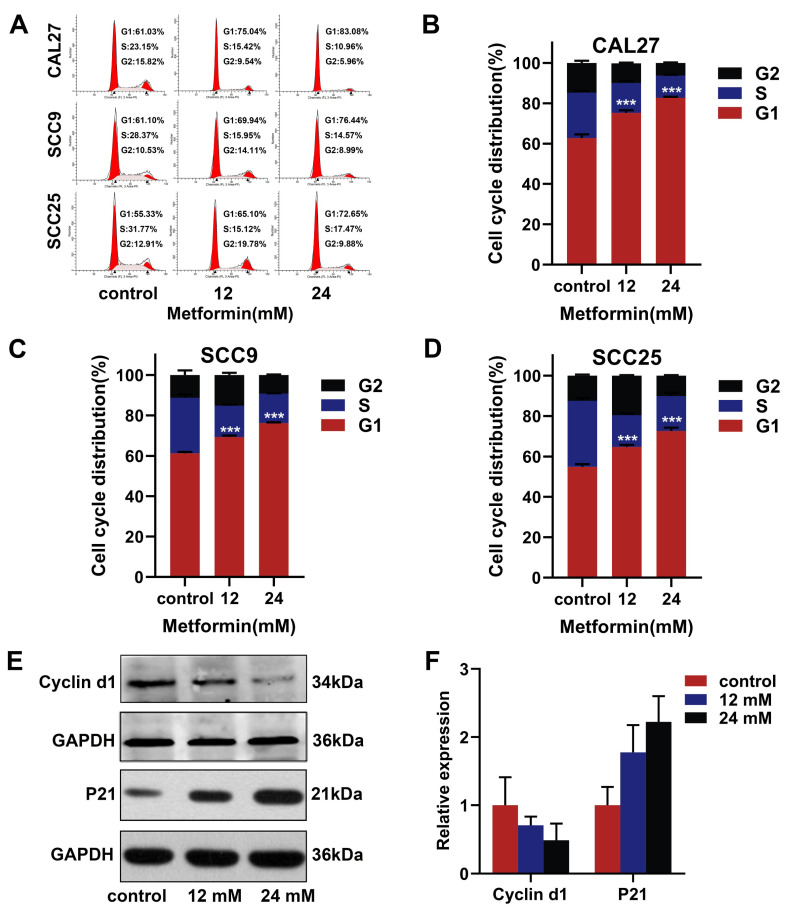
Metformin induced G1 phase cell cycle block in OSCC cells. (**A**–**D**) After treatment with various concentrations of metformin for 48 h, flow cytometry assays were performed to assess the impact of metformin on the cell cycle distribution of OSCC cell lines (CAL27, SCC9, SCC25). (**E**,**F**) CAL27 cells were treated with different concentrations of metformin for 48 h, and, then, the cell cycle related proteins were analyzed by western blot. Original blots see Supplementary Figure S4. GAPDH was used as the internal reference protein. The data are shown in the bar graph as mean ± SD, *** *p* ≤ 0.001 versus control group.

A cell cycle-assay was performed to assess the cell-cycle distribution of OSCC cells treated with metformin. The number of OSCC cells in the G1 phase increased as the concentration of metformin increased (Figure 3A−D). The expression of cell-cycle related proteins was detected by western blot. We found that metformin increased the P21 expression and decreased the Cyclin D1 expression (Figure 3E,F). Overall, metformin induced the G1 phase cell-cycle block in OSCC cells.

### 3.4. Metformin Promotes Apoptosis in OSCC Cells via a Non-ROS-Dependent Pathway

**Figure 4:**
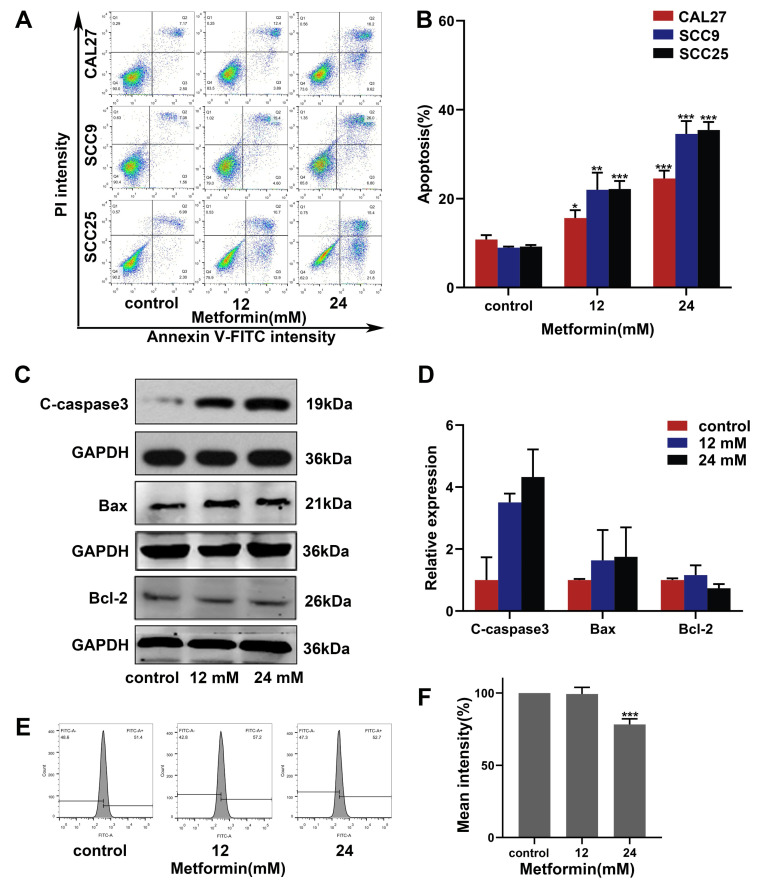
Metformin induced apoptosis in OSCC cells via a non-ROS dependent pathway. (**A**,**B**) The apoptosis assays were performed to assess the impact of metformin on apoptosis of OSCC cell lines (CAL27, SCC9, SCC25). The early apoptotic cells are located in the lower right quadrant, the late apoptotic cells are located in the upper right quadrant, and the necrotic cells are located in the upper left quadrant. (**C**,**D**) CAL27 cells were treated with different concentrations of metformin for 48 h, and then the apoptosis related proteins were analyzed by western blot. Original blots see Supplementary Figure S4. GAPDH was used as the internal reference protein. (**E**,**F**) Flow cytometry was used to measure intracellular ROS levels in CAL27 cells treated with various concentrations of metformin. The data are shown in the bar graph as mean ± SD, * *p* ≤ 0.05, ** *p* ≤ 0.01 and *** *p* ≤ 0.001 versus control group.

**Figure 5:**
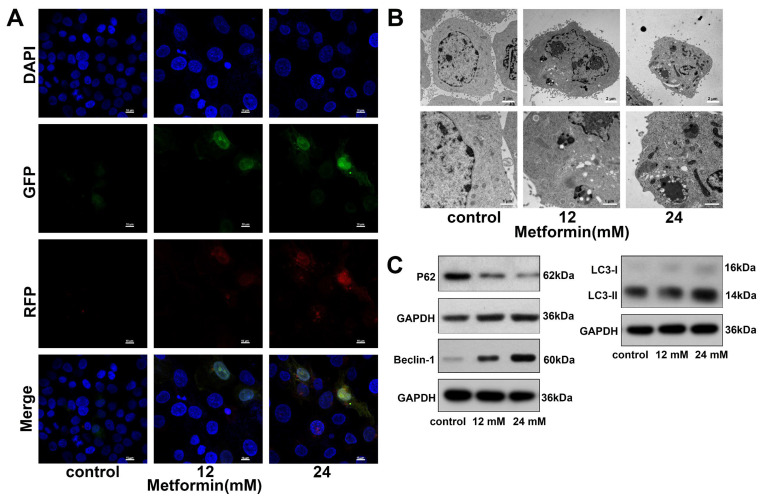
Metformin promoted autophagy in OSCC cells. (**A**). CAL27 cells were treated with various concentrations of metformin for 48 h. Meanwhile, the mRFP-GFP-LC3 adenovirus was used to detect the autophagic flux in CAL27 cells. The yellow dots (RFP+GFP+) were autophagosomes, whereas the red dots were (RFP+GFP-) were autolysosomes. The magnification = 1000×, scale bars = 10 μm (**B**). The autophagosomes and nucleus were observed by the TEM. CAL27 cells were treated with different concentrations of metformin for 48 h. Scale bars = 2 μm and 1 μm (**C**). CAL27 cells were treated with different concentrations of metformin for 48 h, and the autophagy-related proteins were analyzed by western blot. Original blots see Supplementary Figure S4. GAPDH was used as the internal reference protein.

We performed an apoptosis assay to detect the effect of metformin on the OSCC cell apoptosis level. Metformin significantly increased the apoptosis level of OSCC cells (Figure 4A,B). CAL27 cells treated with metformin were observed by TEM. The results of TEM indicated that CAL27 cells treated with metformin showed the characteristics of apoptosis, such as cell shrinkage, nucleoplasm condensation and nuclear fragmentation (Figure 5B). Furthermore, the results of western blot were consistent with the experiments above. The expressions of the pro-apoptotic protein Bax and the apoptotic executive protein Cleaved-caspase3 were upregulated. The expression of anti-apoptotic protein Bcl-2 was downregulated (Figure 4C,D). We performed a ROS assay to verify whether metformin promotes apoptosis in OSCC cells via the ROS-dependent pathway. Surprisingly, the ROS level decreased as the concentration of metformin increased (Figure 4E,F). Collectively, metformin promoted apoptosis in OSCC cells via a non-ROS-dependent pathway.

### 3.5. Metformin Promotes Autophagy in OSCC Cells

We transfected the mGFP-RFP-LC3 adenovirus into CAL27 cells to detect the autophagy level in CAL27 cells. As the concentration of metformin increased, the number of red dots (autophagolysosomes) and yellow dots (autophagosomes) increased (Figure 5A). The autophagosomes in CAL27 cells were directly observed by TEM. As shown in Figure 5B, compared to the control group, metformin significantly increased the number of autophagosomes in CAL27 cells. Finally, we detected autophagy-related proteins by western blot. Metformin increased the ratio of LC3B-II/LC3B-I. Furthermore, after being treated with metformin, the expression of Beclin-1 was upregulated, and the expression of P62 was downregulated (Figure 5C and Figure S1). Taking all these data together, metformin promoted autophagy in OSCC cells.

### 3.6. Inhibiting Autophagy with HCQ Increases the Apoptosis of OSCC Cells Induced by Metformin

**Figure 6:**
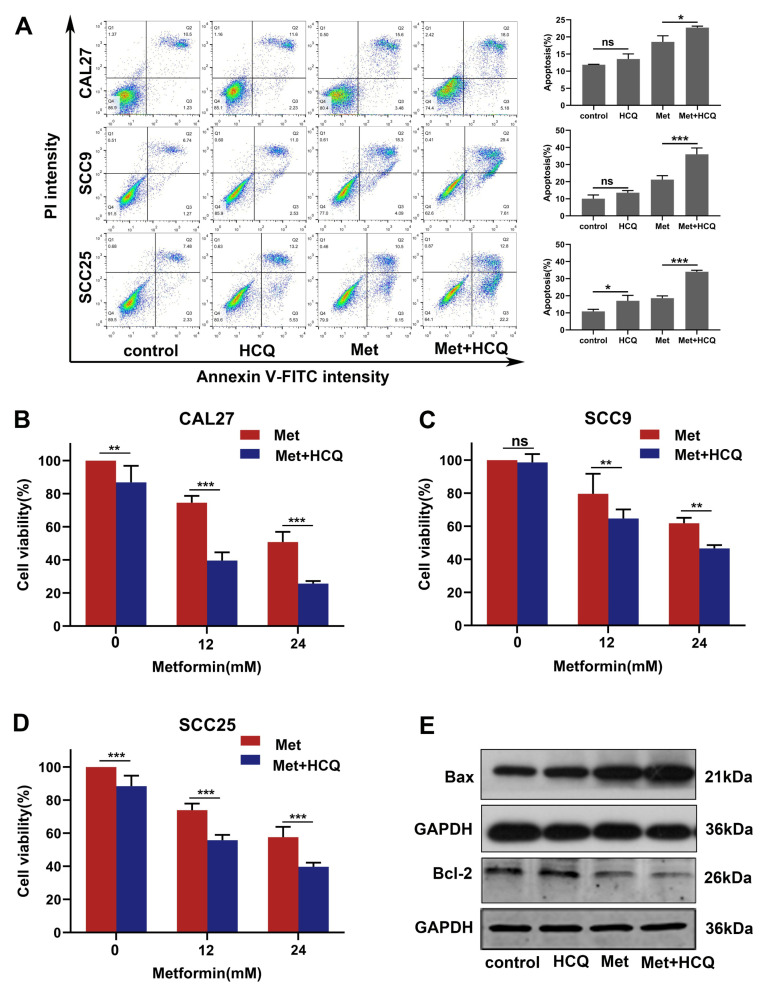
Inhibiting autophagy with HCQ increased the apoptosis of OSCC cells caused by metformin. (**A**) The OSCC cell lines (CAL27, SCC9, SCC25) were treated with metformin (12 mM), HCQ (20 μM), and combined treatment (metformin and HCQ) for 48 h. Flow cytometry assays were performed to assess the apoptosis level in OSCC cells. (**B**–**D**) The OSCC cell lines were treated with various concentrations of metformin (0, 12, 24 mM), with or without HCQ (20 μM), for 24 h. The cell viability was detected by CCK-8 assay. (**E**) CAL27 cells were treated as Figure 6A, and then the apoptosis related proteins were analyzed by western blot. Original blots see Supplementary Figure S4. GAPDH was used as the internal reference protein. The data are shown in the bar graph as mean ± SD. * *p* ≤ 0.05, ** *p* ≤ 0.01, *** *p* ≤ 0.001 and ^ns^ *p* > 0.05. Metformin group and HCQ group versus control group, metformin + HCQ group versus metformin group.

To investigate the interactions between autophagy and apoptosis induced by metformin, the autophagy inhibitor HCQ was used to suppress the autophagy induced by metformin. Flow cytometry analysis revealed that HCQ could enhance the apoptosis induced by metformin in OSCC cells (Figure 6A). HCQ alone had little inhibition on the OSCC cell viability, but it could significantly enhance the inhibition of metformin on the OSCC cell viability (Figure 6B–D). Afterwards, we used western blot to detect the proteins involved in apoptosis. Compared to metformin alone, the combined group significantly overexpressed the Bax and downregulated the Bcl-2 (Figure 6E and Figure S2). Collectively, inhibiting autophagy with HCQ increased the apoptosis of OSCC cells induced by metformin.

### 3.7. Metformin and HCQ Synergistically Suppress OSCC Growth In Vivo

**Figure 7:**
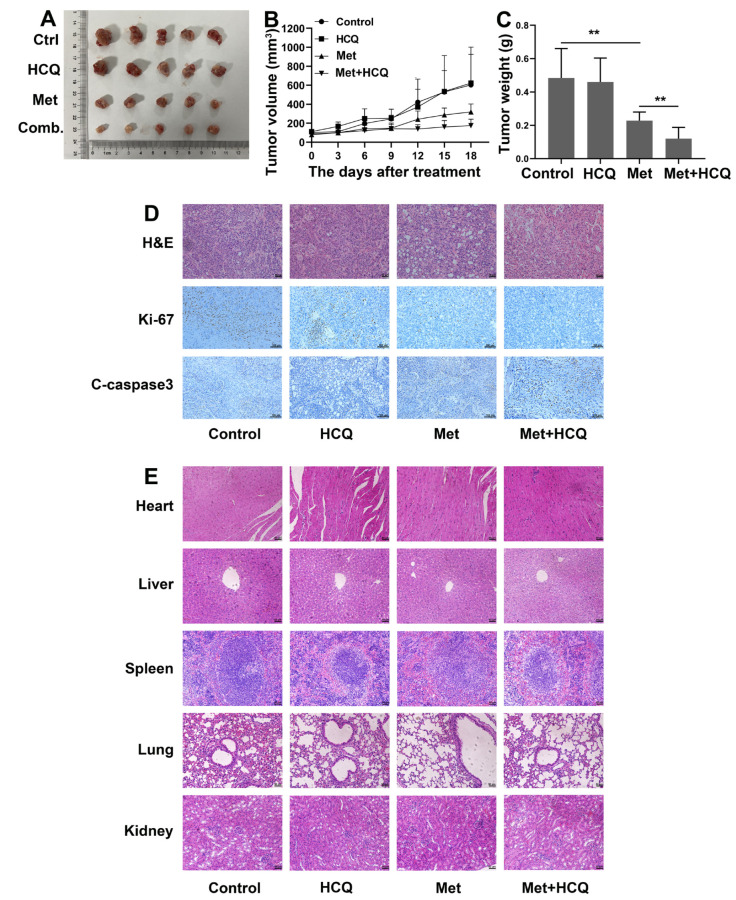
Metformin and HCQ synergistically suppressed OSCC growth in vivo. (**A**) CAL27 cells (1 × 10^7^ cells) were injected into the subcutaneous area of female nude mice. After treatment with PBS, metformin (250 mg/kg/d), HCQ (50 mg/kg/d), and combined treatment (metformin and HCQ) for 18 days, all animals were sacrificed. (**B**) The tumor volume was measured every three days after treatment. (**C**) The tumor weight was measured after all mice were sacrificed. (**D**) The expression of Ki-67 and Cleaved-caspase3 in tumor tissue was analyzed by IHC. The magnification = 100×, scale bars = 100 μm (**E**) H&E staining was conducted to assess the toxicity to the major organ after treatment. The magnification = 200×, scale bars = 50 μm. The data are shown in the bar graph as mean ± SD. ** *p* ≤ 0.01. Metformin group versus control group, the combined group versus metformin group.

The xenograft transplantation model of OSCC was established to evaluate the anti-OSCC effect of metformin and HCQ in vivo (Figure S3). The results of animal experiments were consistent with the results in vitro. Metformin could inhibit the growth of OSCC in vivo, and HCQ could enhance the anti-growth effect of metformin (Figure 7A). After 18 days of treatment, we removed the tumors from the mice. As shown in Figure 7B,C, the tumor volume and the tumor weight of the metformin group and the combined group were smaller than the control group, especially the combined group. The IHC was used to detect the Ki-67 and Cleaved-caspase3 expression levels in tumor tissues. As shown in Figure 7D, after treatment with metformin, the expression of Ki-67 was significantly downregulated and the expression of Cleaved-caspase3 was upregulated, especially when combined with HCQ. The results of H&E staining showed that there was no obvious vital organ damage in all groups (Figure 7E). Collectively, metformin and HCQ synergistically suppressed OSCC growth in vivo at a safe dose.

## 4. Discussion

OSCC ranks sixth among all malignant tumors worldwide, which accounts for more than 90% of malignant tumors in the head and neck [26]. The main risk factors of OSCC include smoking, alcohol abuse, betel quid chewing and viral infections [27]. Currently, surgery and chemotherapy are still the first-line regimens for OSCC. However, surgical treatment has the high risk of tissue damage and is likely to cause serious oral deformities and functional disorders [28]. In addition, the effectiveness of chemotherapeutic agents is limited by toxicities and drug resistance [29]. Therefore, a safe and effective treatment for OSCC is urgent. In 2005, Evans et al. found that metformin was able to reduce cancer incidence in type II diabetes patients for the first time [30]. Subsequently, preclinical studies of metformin as an antitumor drug gradually increased. ZHENG et al. found that metformin inhibited NF-κB and STAT3 signaling pathways by activating AMPK, thereby inhibiting the growth of HCC cells [31]. In addition, there are more than 50 IV phase clinical trials about metformin as a cancer treatment drug in the US clinical trial database (http://clinicaltrials.gov/) (accessed on 5 February 2022). Metformin, currently the first-line drug for type II diabetes, is affordable and safe. Therefore, metformin has great prospects as a therapeutic drug for OSCC.

The uncontrolled cell cycle is one of the hallmarks of tumorigenesis, and inhibition of the cell cycle is the action mechanism of many anti-tumor drugs [32]. The G1 checkpoint determines the transition of the G1/S phase, which is critical in cell proliferation [33]. The results of our study showed that metformin arrested OSCC cells in the G1 phase. P21 belongs to the CIP/KIP family [34] and is a critical cell cycle inhibitory protein, which arrests the cells in the G1 or G2 phase by inhibiting the Cyclin/CDK complex. Cyclin D1 can combine with CDK2 and CDK6 to form a complex, which drives cells from the G1 phase to the S phase and promotes cell proliferation [35]. We proved that metformin could upregulate the P21 expression and downregulate the Cyclin D1 expression in OSCC cells. Overall, the results above confirm that metformin arrests OSCC cells in the G1 phase.

Migration and invasion are the characteristics of malignant tumors, and tumor recurrence is due to tumor migration and invasion. The results of our study showed that metformin inhibited the migration and invasion of OSCC cells in a dose-dependent manner. Migration and invasion in OSCC cells involve complex multi-step processes that are regulated by multiple genes. The specific mechanism by which metformin inhibits migration and invasion still needs further study.

Apoptosis is a form of PCD controlled by genes, which is autonomous and orderly [15]. Most anti-tumor drugs can induce apoptosis to suppress the progression of tumors. After the activation of apoptosis, the integrity of the mitochondrial membrane is damaged, and mitochondrial function is disordered. The cytochrome-*C* transported into the cytoplasm triggers the caspase cascade reaction (especially caspase-3), which eventually results in apoptosis [36]. The Bcl-2 protein family (Bax and Bcl-2) plays a significant role in regulating mitochondrial membrane integrity and controlling the apoptosis. Bax promotes apoptosis by disrupting mitochondrial membrane integrity, while Bcl-2 inhibits apoptosis by maintaining mitochondrial membrane integrity [37]. The results of flow cytometry showed that the apoptosis rate was increased as the concentration of metformin increased. After treatment with metformin, we could observe the apoptotic morphology changes in OSCC cells, including cell shrinkage, nuclear fragmentation and nucleoplasm condensation. Furthermore, the expression of Cleaved-caspase3 and Bax were upregulated, and the expression of Bcl-2 was downregulated. We conclude that metformin promotes OSCC cell apoptosis in a caspase-dependent manner. The main sources of ROS are NADPH oxidase (NOX) and mitochondria, and excessive ROS can promote apoptosis [38]. Previous studies have shown that metformin can induce ROS production to promote tumor apoptosis, but other studies have shown that metformin can promote breast cancer apoptosis via a ROS-independent pathway [39,40]. Surprisingly, we found that the ROS level of OSCC cells was reduced after being incubated with metformin. These data above suggest that metformin promotes OSCC apoptosis in a ROS-independent mechanism, but whether metformin promotes apoptosis by reducing ROS needs more studies.

Autophagy is a lysosome-mediated cellular degradation process in eukaryotic cells, and intracellular proteins and organelles enter lysosomes to be broken down and digested [20,21]. Autophagy is a double-edged sword in tumors, playing opposite roles in different stages of tumors. In the early stages of tumors, autophagy can inhibit tumor progression, and, in the late stages, autophagy is a tumor-promoting factor [22]. Our data showed that metformin could enhance the autophagy flow of OSCC cells. Furthermore, we directly observed the increase of autophagosomes in OSCC cells treated with metformin by TEM. When autophagy occurs, the LC3 localized in cytoplasm (LC3-I) is covalently bound with phosphatidylethanolamine to generate LC3 II, which is bound to autophagosomes [41]. We found that metformin could increase the ratio of LC3II/LC3I. Beclin-1 is bound to class 3 PI3K kinase (Vps34) to form a complex, which is important to initiate autophagy [42]. P62 is an autophagic substrate, the expression of which is inversely related to autophagic flow [43]. In this study, we found that metformin downregulated the expression of P62 and upregulated the expression of Beclin-1. The data above suggest that metformin promotes the autophagy of OSCC cells. HCQ is an autophagy inhibitor that increases the pH of lysosomes, thereby inhibiting autophagosome–lysosome fusion and lysosomal protein degradation [44]. In our study, HCQ was used to inhibit autophagy to explore the influence of metformin-induced autophagy on the progression of OSCC. Our data suggested that the anti-proliferation effect of metformin on OSCC cells was enhanced after autophagy was inhibited. Therefore, we speculated that autophagy induced by metformin was a tumor-promoting factor. Autophagy and apoptosis are critical for maintaining cellular homeostasis, and there is a complex interaction between them. The anti-apoptotic protein Bcl-2 binds to the BH3 domain of the autophagy key protein Beclin-1, which is the molecular basis for the crosstalk between autophagy and apoptosis [45]. Our findings suggested that inhibition of autophagy enhanced metformin-induced apoptosis by upregulating the expression of Bax and downregulating the expression of Bcl-2. Finally, this conclusion was proved in vivo. We found that autophagy inhibitor HCQ enhanced the anti-OSCC effect of metformin in vivo without side effects.

## 5. Conclusions

**Figure 8:**
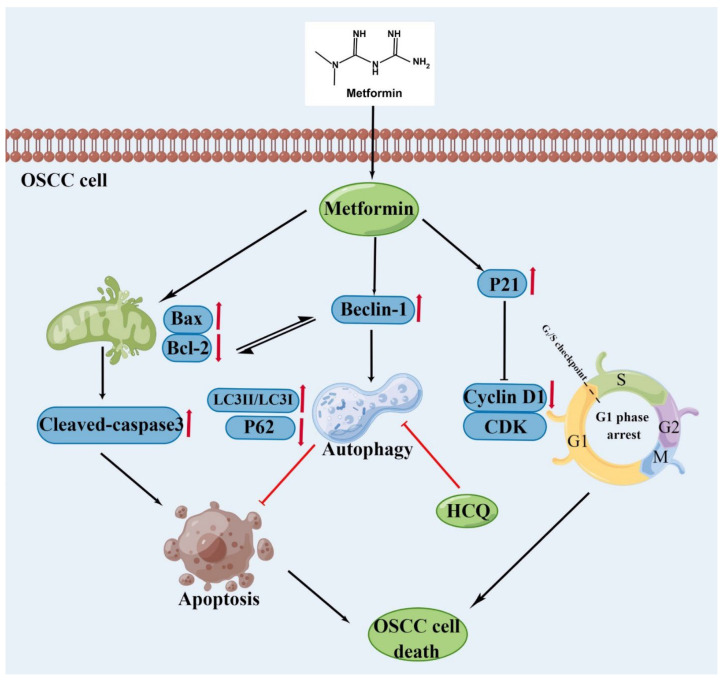
Biochemical pathways involved in the effects of metformin treatment on Oral Squamous Cell Carcinoma (OSCC) cells. Metformin suppresses the proliferation of OSCC cells via the induction of cell cycle arrest and apoptosis. Inhibition of metformin-induced autophagy with HCQ increases the apoptosis of OSCC cells. The figure was created by the Figdraw.

To sum up (Figure 8), the results firstly demonstrated that metformin inhibited the proliferation of OSCC cells and arrested tumor cells in the G1 phase. Secondly, metformin also inhibited the migration and invasion ability of OSCC cells. Finally, metformin promoted apoptosis and autophagy in OSCC cells, and inhibition of autophagy could enhance the anti-OSCC effect of metformin. Therefore, it is highlighted that a combination of autophagy inhibitor HCQ and metformin might be a promising therapeutic strategy for OSCC.

## Data Availability

All datasets generated for this study are included in the article/Supplementary Material.

## Supplementary Materials

The following supporting information can be downloaded at: https://www.mdpi.com/article/10.3390/cancers14174185/s1, Figure S1: Statistical histogram of gray value of autophagy related protein. Figure S2: Statistical histogram of gray value of apoptosis related protein. Figure S3: The timeline of the animal experiment. Figure S4. Original Images for Blots/Gels.
