# Supplementary material for: Inhibition of Autophagy Promotes the Anti-Tumor Effect of Metformin in Oral Squamous Cell Carcinoma
Source: Cancers (Basel). 2022 Aug 29;14(17):4185. doi: 10.3390/cancers14174185 (PMC9454503; doi:10.3390/cancers14174185)
Supplement: Supplementary file 1 [file cancers-14-04185-s001.zip › cancers-1880141-supplementary.pdf]

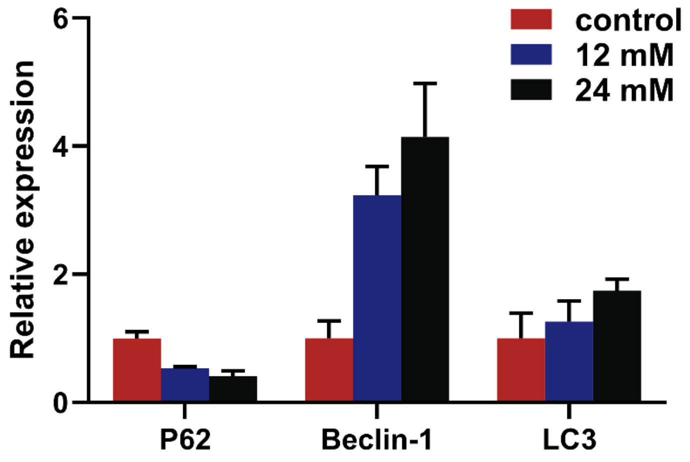

Figure S1. Statistical histogram of gray value of autophagy related protein.

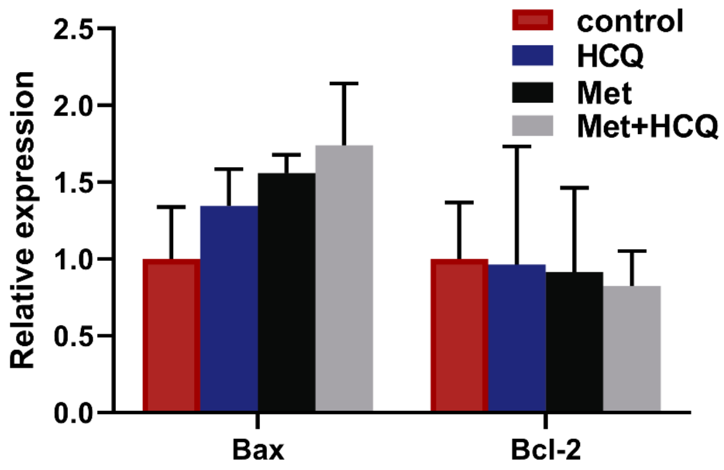

Figure S2. Statistical histogram of gray value of apoptosis related protein.

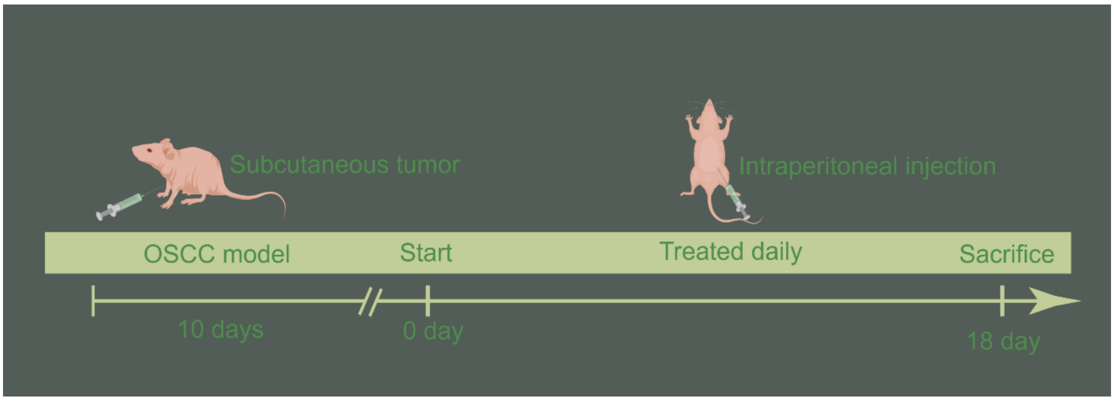

Figure S3. The timeline of the animal experiment. The figure was created by the Figdraw.

Figure S4. Original Images for Blots/Gels

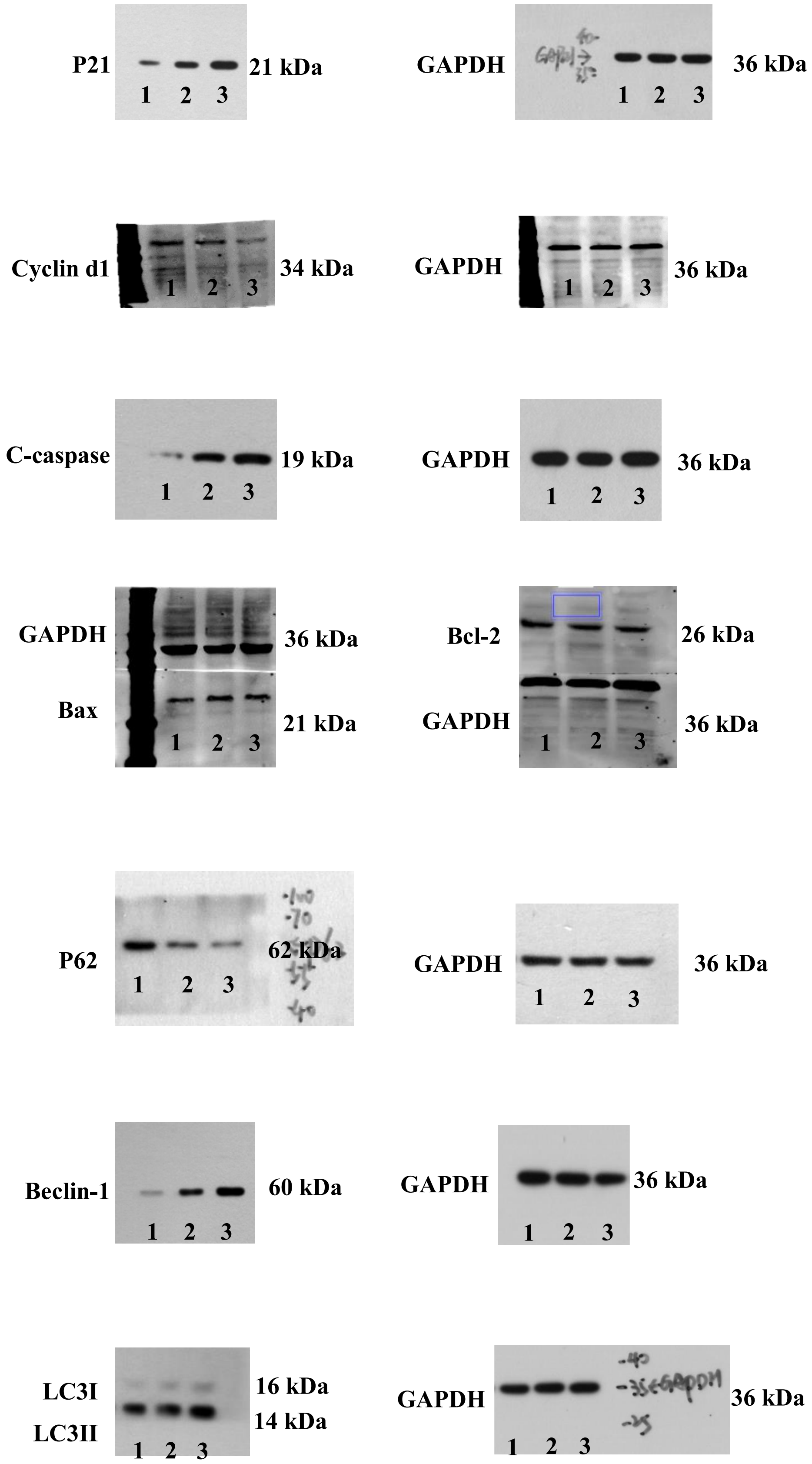

1: control; 2: 12 mM; 3: 24 mM

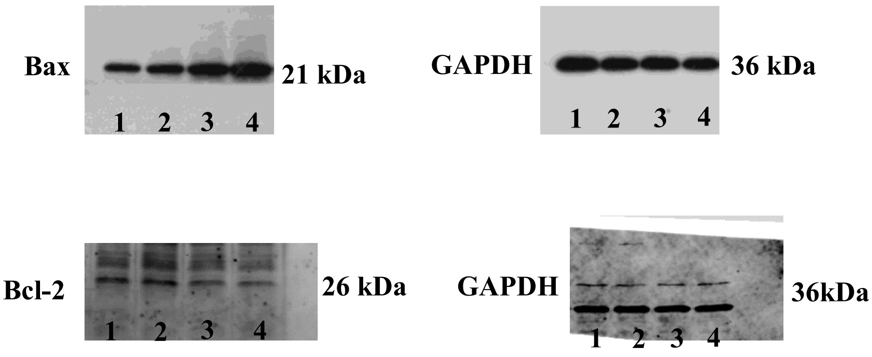

1: control; 2: HCQ; 3: Met; 4: Met+HCQ
